# Supplementary figures and images for: Bioinformatics analysis of laryngeal squamous cell carcinoma: seeking key candidate genes and pathways
Source: PeerJ. 2021 Apr 14;9:e11259. doi: 10.7717/peerj.11259 (PMC8052978; doi:10.7717/peerj.11259)

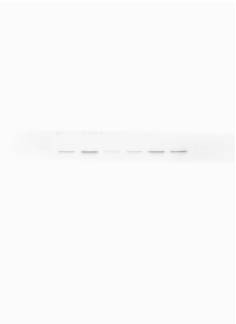

Supplement: Supplemental Information 9 [file peerj-09-11259-s009.zip › Western/MMP1.jpg]

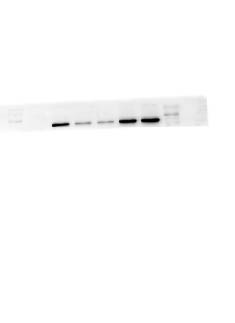

Supplement: Supplemental Information 9 [file peerj-09-11259-s009.zip › Western/SERPINE1.jpg]

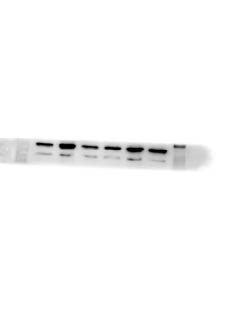

Supplement: Supplemental Information 9 [file peerj-09-11259-s009.zip › Western/SPP1.jpg]

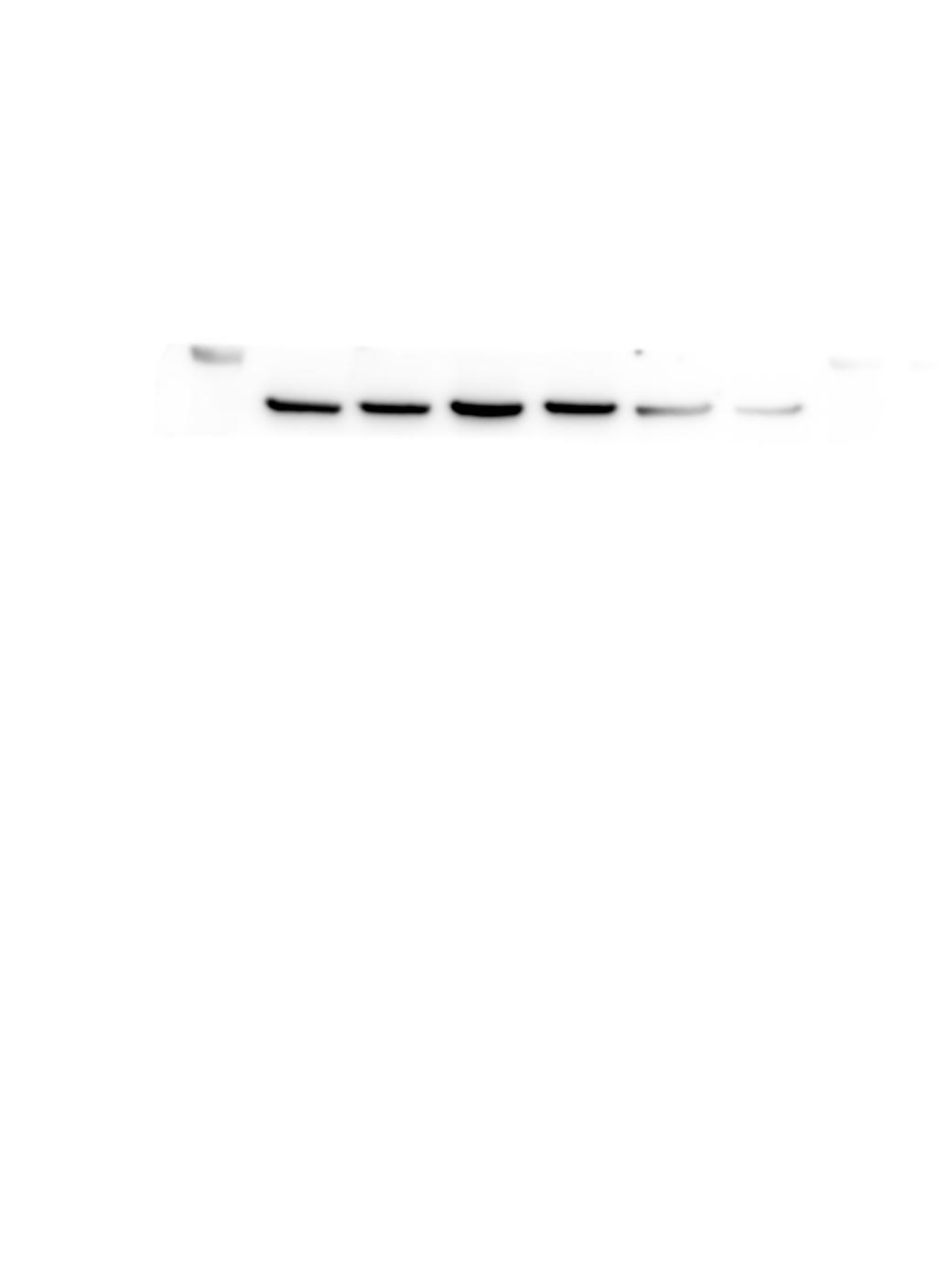

Supplement: Supplemental Information 9 [file peerj-09-11259-s009.zip › Western/β-actin.jpg]
